# Enhancing the Survival of Ichneumonid Parasitoid *Campoletis chlorideae* (Hymenoptera: Ichneumonidae) by Utilizing Haserpin-e Protein to Effectively Manage Lepidopteran Pests

**DOI:** 10.3390/insects16050474

**Published:** 2025-04-29

**Authors:** Liuming Huo, Xue Yao, Ningbo Zhang, Shengyi Wang, Sufen Bai, Yanmei Wang, Jizhen Wei, Shiheng An

**Affiliations:** 1 College of Forestry, Henan Agricultural University, Zhengzhou 450046, China; 13253830925@163.com; 2 College of Plant Protection, Henan Agricultural University, Zhengzhou 450046, China; yaoxue983@163.com (X.Y.); zhangningbo0109@163.com (N.Z.); wsywsywsy1478@163.com (S.W.); sfbai68@126.com (S.B.); anshiheng@aliyun.com (S.A.)

**Keywords:** *Helicoverpa armigera*, indoor breeding, innate immune

## Abstract

The ichneumonid parasitoid *Campoletis chlorideae* is an important natural enemy of lepidopteran pests in different agro-ecosystems, specifically targeting early larvae (second- and third-instar). Enhancing the survival of *C. chlorideae*, especially within hosts, remains a significant technical challenge for large-scale indoor reproduction. This study investigates the use of endogenous serpin-e protein, derived from the host *Helicoverpa armigera* (Haserpin-e), to improve the survival rate of *C. chlorideae* in indoor reproduction. The results demonstrated that Haserpin-e protein significantly enhanced cocoon production in *C. chlorideae*, with no observable adverse effects on the life history traits of both F0 and F1 generations of *C. chlorideae*. By investigating the mechanism underlying cocoon formation promotion, it was found that Haserpin-e protein reduced the encapsulation, inhibited melanization, as well as suppressed the expression of antimicrobial proteins (AMPs) in *H. armigera*. This study provides novel insights into improving the survival of *C. chlorideae* by inhibiting host immune responses through the application of its endogenous Haserpin-e protein during large-scale indoor reproduction efforts. Additionally, this research further elucidates the multifaceted functionality of Haserpin-e proteins by demonstrating their role in regulating innate immune processes in *H. armigera*, including negatively regulating encapsulation, melanization, and AMP expression.

## Simple Summary

Enhancing the survival of the ichneumonid parasitoid *Campoletis chlorideae* remains a significant technical challenge for large-scale indoor reproduction. This study develops a novel methodology to promote cocoon formation in *C. chlorideae* by utilizing the host protein Haserpin-e to inhibit the insect’s innate immune responses. To elucidate the mechanism underlying enhanced cocoon formation, we found that Haserpin-e protein reduced encapsulation, inhibited melanization processes, and suppressed the expression of antimicrobial proteins (AMPs) in the host *Helicoverpa armigera*. Furthermore, this study extends our understanding of the functionalities of Haserpin-e proteins.

## 1. Introduction

*Campoletis chlorideae* Uchida (Hymenoptera: Ichneumonidae) is an important natural enemy of more than 30 Lepidopteran pest species, such as *Helicoverpa armigera* Hübner, *Spodoptera litura* Fabricius, *Spodoptera frugiperda* Smith, *Agrotis ipsilon* Hufnagel, *Mythimna separata* Walker, and so on [1,2,3,4,5]. This parasitoid specifically targets early larvae (second- and third-instar) of the Lepidopteran species, exerting significant control over the population size of these pests in agricultural fields. A study in Hunan province, China, reported that this parasitoid’s parasitism rate on *H. armigera* larvae ranged from 25.1% to 63.1% [3]. Furthermore, it has shown a notable efficacy in reducing pest numbers by 20% through parasitism on plants, achieving an impressive 80.5% parasitization rate on *H. armigera* larvae in chickpea fields [6,7]. Consequently, *C. chlorideae* is recognized worldwide as a promising biological control agent for managing Lepidopteran pest populations.

Despite the high efficacy of *C. chlorideae* in controlling Lepidopteran pests, the survival capacity after parasitism remains a significant challenge for the mass-rearing of *C. chlorideae* due to the strong immune responses elicited by the host. The survival of parasitoids depends on their ability to overcome the host’s immunological rejection. Endoparasitic insects develop as larvae within the host’s body cavity, where both the oviposited eggs and the developing larvae have evolved various strategies to evade or suppress host immune responses, manipulate host immunity, and regulate their growth and development [8]. On the one hand, *C. chlorideae* disrupts cellular immune responses by interfering with apoptosis [9,10], inactivating hemocytes [11], degrading the cytoskeleton [12], and disrupting encapsulation [13,14]. On the other hand, it inhibits humoral immune responses by suppressing melanization [15,16] and reducing the production of defense molecules such as antimicrobial peptides (AMPs) [17].

Most of the host’s immune responses are classified as innate immunity. Insects employ innate immunity as their primary defense mechanism against pathogen attacks. Serpins within insects negatively regulate cascades of serine protease activation triggered by innate responses, which encompass hemocyte clotting, melanization, the hemocyte-mediated melanotic encapsulation response, and the expression of AMPs [18,19]. Normally, host encapsulation occurs after *C. chlorideae* lays eggs within the host’s body. Host encapsulation is subsequently followed by melanization, a process that effectively kill the exogenous parasite. Melanization, a rapid localized blackening response activated by phenol oxidase (PO), occurs at wound sites and in response to foreign objects such as parasitic wasp eggs [20]. During this process, polyphenol oxidase (PPO) zymogen is converted into active PO by a clip domain serine proteinase [21,22]. The resulting active PO catalyzes the oxidation of phenols to form quinones, which then spontaneously polymerize to generate melanin [23,24]. This process is tightly regulated by serpins that specifically target certain serine proteases responsible for melanization regulation [19]. Several serpins have been identified as melanization regulators in insects, such as SRPN1 and SRPN2 in *Aedes agypti* [25]; serpin-5 and serpin-9 in *H. armigera* [26]; SRPN2 in *Anopheles gambiae* Meigen [27], serpin-5, -6, -15, and -32 in *B. mori* [28,29,30,31]; serpin-1, serpin-3, serpin-4, serpin-5, serpin-6, serpin-7, serpine-9, serpine-12, and serpine-13 in *Manduca sexta* Blanchard [32,33,34,35]; SPN40, SPN48, and SPN55 in *Tenebrio molitor* Linnaeus [36], and serpin-4 in *Ostrinia furnacalis* Guenée [37].

Previously, we have identified a serpin protein in *H. armigera* (Haserpin-e) that exhibits inhibitory activity against midgut serine proteases [38,39]. However, it is not known whether Haserpin-e negatively regulates the serine protease cascade triggered by innate immune responses, thereby improving the survival of *C. chlorideae* in the host. If Haserpin-e could improve the survival of *C. chlorideae*, this will facilitate the mass-rearing of *C. chlorideae* and contribute to its application as a biocontrol agent for managing Lepidopteran pest populations. In this study, we first examined the impact of Haserpin-e on the cocoon formation rate of *C. chlorideae* (where cocoon formation indicates that *C. chlorideae* successfully completed its growth and development within the host). Subsequently, we assessed the effects of Haserpin-e on the life history traits of *C. chlorideae*, including cocoon formation rate, cocoon size and adult size, sex ratio, mating rate, adult survival time, mature eggs, and number of ovarioles, and these life history traits of F1 generation. These factors are critical in determining the quality of *C. chlorideae* and ensuring sustainable field reproduction. Finally, we explored the functions and mechanisms of Haserpin-e in *H. armigera* and the cocoon formation promotion process. This research aims to develop a new methodology for the large-scale propagation of *C. chlorideae*, thereby facilitating efficient biological control of Lepidopteran pests.

## 2. Materials and Methods

### 2.1. Insects and Haserpin-e

The *H. armigera* larvae were obtained from Henan Jiyuan Baiyun Industry Co., Ltd. (Jiyuan, China) and reared in the laboratory using an artificial diet free of insecticides, following previously established protocols [40].

The *C. chlorideae* colony, originally collected from the Xuchang Campus of Henan Agricultural University on 7 June 2023, has been maintained in the laboratory for multiple generations by rearing on *H. armigera* larvae at a controlled temperature of 25 ± 1 °C, 55% humidity, and photoperiod of 16 h light and 8 h dark [41].

The Haserpin-e expression vector was obtained from Zhang et al. [38] and the Haserpin-e protein was expressed and purified by Hangzhou Huanan Biotechnology Co., Ltd. (Hangzhou, China). The Haserpin-e protein was produced using the Haserpin-e-pet-28a (+) expression plasmid in *Escherichia.coli* expression Rosetta (DE3) strain. After culturing, the bacterial cells were induced with IPTG (Isopropyl-β-D-thiogalactoside) to express the raw Haserpin-e protein. Subsequently, the raw protein was extracted through ultrasonic disruption and centrifugation, followed by resuspension. Gradient dialysis with urea was performed using a nickel column, and the protein was finally concentrated using an ultrafiltration tube and dissolved in phosphate-buffered saline (PBS). The final protein concentration was determined by the Bradford method.

### 2.2. Effect of Haserpin-e on C. chlorideae

The artificial diet of *H. armigera* was prepared and supplemented with PBS buffer or Haserpin-e proteins (with final concentrations of 6 or 10 μg/g Haserpin-e proteins). Previous experiments have demonstrated that these two concentrations (6 and 10 μg/g) can effectively enhance the cocoon formation rate; however, the cocoon formation rate does not continue to increase with higher concentrations of Haserpin-e. On the one hand, 480 (120 *H. armigera* for each treatment, 30 *H. armigera* for each repetition in each treatment) early second-instar *H. armigera* were prepared. And these *H. armigera* were provided with diets corresponding to the treatment concentration for a duration of 24 h prior to parasitism. After eating Haserpin-e or PBS, each of the *H. armigera* would be parasitized. On the other hand, sixty successfully mated female *C. chlorideae* adults, aged 5–7 days (this stage exhibits the greatest reproductive capacity), were selected and used to parasitize the previously treated larvae. *H. armigera* in each treatment (120) were parasitized by 15 *C. chlorideae*, with 8 *H. armigera* individuals parasitized per *C. chlorideae* to ensure that the evaluated parasitoid wasps represent the population characteristics. One *C. chlorideae* was placed in a finger tube (25 mL) and positioned upside down on the table. The tube was quickly lifted, and one *H. armigera* was introduced. The *C. chlorideae* promptly pricked the *H. armigera* (within 5 s), indicating that an egg had been laid inside the *H. armigera*. The parasitized *H. armigera* were fed under laboratory conditions, and the life history traits of F0 generation were recorded in each treatment, including the cocoon production rate after 7 days, the emergence rate after 14 days, cocoon size and adult size, sex ratio, mating success rate, adult lifespan, number of ovarian ducts, and the mature eggs in the dead adults. The size of cocoons was used to indicate the size of pupae, and measurements were taken for both the length and width of the cocoons [42]. Following the emergence of *C. chlorideae*, the cocoons were carefully collected and placed on transparent glass cups. They were then secured with double-sided adhesive tape before being examined under an OLYMPUS-SZX7 microscope for determination. The tibial length of the hind legs was utilized as an indicator of adult size [42]. Tibial segments from the hind feet of naturally deceased *C. chlorideae* were carefully removed with pointed forceps while gently holding the carapace, arranged neatly on a clean glass plate, and then a photograph was taken using a stereoscope and measured their length under the stereoscope. Photographs of *C. chlorideae* cocoons under various experimental treatments were taken using a stereoscope (SZX7, OLYMPUS, Tokyo, Japan). Upon emergence, <24 h *C. chlorideae* female were paired with males. Each pair was placed in a 25 mL cup for 15 min. Successful mating was determined by the sustained wing vibration exhibited by the male and the occurrence of copulation. If the females are not mated on the first day, they will be provided with additional opportunities for mating on the following days using the aforementioned methods. Finally, the number of mated female adults was recorded for each treatment group. The adults *C. chlorideae* were reared in 25 mL cups and provided with 10% honey-soaked cotton balls placed inside the cup (replaced daily) within a temperature-controlled incubator set at 26 ± 1 °C and 55% humidity until their demise. Five to seven days after the above *C. chlorideae* adults emerged, 15 mated *C. chlorideae* in each treatment were randomly selected for a total of 60 *C. chlorideae*. These *C. chlorideae* were used to investigate the effects of Haserpin-e or PBS on F1 generation (Figure S1). Each *C. chlorideae* in each treatment parasitized 6 normal *H. armigera* (90 *H. armigera* for each treatment, with 30 *H. armigera* per repetition in each treatment).

To further investigate the specific effects of Haserpin-e on reproduction, we prepared 360 *H. armigera* (90 *H. armigera* for each treatment, with 30 *H. armigera* per repetition in each treatment). Additionally, 60 *C. chlorideae* were used for parasitization (each parasitizing 6 *H. armigera*). The dissection of *C. chlorideae* occurred at the sixth day (the reproductive peak) after their emergence, where the head was firmly pressed with a dissecting needle, while gentle pressure was applied with another dissecting needle on the abdomen of the female wasps to carefully extract the intact ovary tube. Subsequently, they were immersed in a PBS solution with a pH of 7.2. The ovaries were meticulously dissected using dissecting forceps under a stereomicroscope (SZ2-LGB, Shenzhen, China, Olympus Corporation of Japan), and then placed on slides for the quantification of ovarian tubes the using an OLYMPUS-SZX7 microscope. After that, the oviducts were broken to count the number of mature eggs inside (Figure S1).

The second-instar *H. armigera* is the most suitable parasitic stage, and with the increase in instar the host immunity is enhanced and the survival rate of *C. chlorideae* decreases [43]. In order to further verify the effect of Haserpin-e on promoting cocoon formation and prolonging parasitic time for convenient breeding, we further verified the effect of Haserpin-e at the instar for 1 day and 2 days using 360 3rd (1 day or 2 days) *H. armigera* (90 *H. armigera* for each treatment, with 30 *H. armigera* per repetition in each treatment). Additionally, 60 *C. chlorideae* were used for parasitization (each parasitizing 6 *H. armigera*). The cocoon production rate and the emergence rate were investigated (Figure S1).

### 2.3. Effect of Haserpin-e on Encapsulations of Host

A total of 600 early second-instar *H. armigera* larvae were prepared (150 for each treatment, with 50 as a replication per treatment) and treated with either PBS or Haserpin-e prior to being monoparasitized by *C. chlorideae* (Figure S1). A total of 60 *C. chlorideae* were used to parasitize, and each one parasitized 6 *H. armigera* (Figure S1). After incubation at 34 °C for 48 h, the *H. armigera* were dissected under a dissecting microscope (SOPTOP s1200-3, Yuyao, China, Ningbo Sunny Instruments Co., Ltd.) to observe encapsulation. The encapsulation rate was calculated as the number of capsules in each group divided by the total number of embryos.

### 2.4. The Effect of Haserpin-e on Hemolymph In Vitro

Hemolymph extraction: One hundred third-instar *H. armigera* were chilled on ice for 5 min to prepare them. Hemolymph fluid was collected using the aforementioned method. The collected hemolymph was then subjected to centrifugation at 12,000 rpm for 10 min at 4 °C, followed by collection of the supernatant for subsequent testing. For the following experiments, three technical repetitions were performed.

Haemolymphatic darkening analysis: The prepared hemolymph was mixed with Haserpin-e (final concentration of 0.2 μg/μL). The control group was treated with an equal volume of PBS. Subsequently, the samples were thoroughly mixed and sealed with a film. It was kept at room temperature. At 0 h, 24 h, 48 h, 72 h, and 120 h time points, 1.5 μL of the solution was extracted and deposited onto a glass slide for photography. After each usage, it was resealed with the film until the completion of photography at 120 h.

The method for determining the activity of the PO enzyme was adapted from a previous study [44]. Hemolymph samples were prepared by mixing with PBS or Haserpin-e (final concentration of 0.2 μg/μL) incubated at room temperature for 10 min. Only hemolymphs without any additives served as the blank control. Subsequently, the PO enzyme activity was determined using a commercially available PO kit (Grace, Suzhou, China), and measured using a microplate reader (BioTEK, Charlotte, VT, USA) at a wavelength of 490 nm.

Following the same procedures as for PO enzyme activity, samples were prepared to measure PPO and trypsin enzyme activities. PPO enzyme activity was analyzed using the PPO enzyme activity kit (Grace, Suzhou, China) according to the manufacturer’s instructions [45]. The absorbance of PPO enzyme activity was measured at a wavelength of 420 nm using a microplate reader. Trypsin enzyme activity was analyzed using the trypsin kit (Grace, Suzhou, China) and measured at a wavelength of 405 nm using a microplate reader [46].

### 2.5. The Impacts of Haserpin-e on Enzyme Activities in Non-Parasitized and Parasitized H. armigera Larvae

A total of 240 *H. armigera* were prepared as the above PBS or Haserpin-e treated (60 for each treatment, 20 as a replication in each treatment) without being parasitized (Figure S1). A total of 240 *H. armigera* (60 for each treatment, 20 as a replication in each treatment) were also prepared as the above PBS or Haserpin-e treated, and these were parasitized once by the *C. chlorideae* (Figure S1). Then, the treated *H. armigera* ate a normal artificial diet for 24 h in 26 ± 1 °C incubator. Later, the *H. armigera* were dissected to remove cephalic shells, the epidermis, and food residue, and the remaining tissues were collected in 1.5 mL tubes. The collected tissues were weighed, and PBS was added at a ratio of 1 mg to 9 mL. Following complete grinding, the tissues were centrifuged at 6000 rpm at 4 °C for 10 min. Subsequently, the supernatant was transferred and further diluted 10 times in PBS, using a BCA protein assay kit (Nanjing, Beijing, China) to test the protein concentration before determining the enzyme activity. Subsequently, the activities of trypsin, PO, and PPO enzymes were quantified using the corresponding enzyme activity kit as described above. Each enzyme activity was tested three times for each sample.

### 2.6. Sample Preparation, cDNA Synthesis and Real-Time Quantitative PCR Analyses

Referring to the above method in Section 2.5, the samples for each treatment were prepared (Figure S1). Then, the total RNA was extracted using RNAiso Plus (TaKaRa, Kusatsu, Japan). The reverse transcription kit HiScript^®^III RT Su-perMix for qPCR (+gDNA wiper) (Vazyme, Nanjing, China) was employed for cDNA synthesis, following the provided instructions. The real-time quantitative PCR (RT-qPCR) reaction system and process, as well as the calculation methods, were performed following the methodology outlined by Wei et al. [47]. All the relative primers of AMPs (cecropin 1, cecropin D, gloverin, and moricin) and the gene ID were listed in Table S1. The reference genes used in this study were *EF-1α* and *β-Actin* of *H. armigera*. Each of the genes expressions were tested three times for each sample.

### 2.7. Statistical Analysis

Two-way and one-way analysis of variance (ANOVA) were used to statistically analyze the above data with SPSS 22.0 at the *p* < 0.05 level of significance. Before analysis of variance, all the data were tested under the assumptions of normality and homogeneity of variance. When the data did not meet the assumptions of normality and homogeneity of variance, the data were transformed by arcsine or log, and then variance analysis was conducted. If all the transformations could not meet the analysis requirements, the two-way non-parametric ANOVA method was used, as seen in Shen et al. [48]. Then, the Tukey method was used for multiple comparisons, and the false discovery rate was used to correct the comparison results. One-way analysis of variance in the life history traits, including cocoon production, cocoon dimensions (length and width), adult size, sex ratio, mating rate, female and male adults’ longevity, ovarian count, and mature egg count, and the activities of trypsin, PO, PPO, encapsulation rate, and AMPs expression among the four treatments (CK, PBS, and 6 and 10 μg/g Haserpin-e) were compared with Tukey’s multiple comparisons test. And the significantly differences in the activities of trypsin, PO, PPO, and TH enzymes among the three treatments (CK, PBS, and Haserpin-e) were compared with Tukey’s multiple comparisons test. The differences in trypsin, PO, PPO, and TH enzyme activities, as well as the AMPs expression between no-parasitized and parasitized *H. armigera* larvae, were analyzed by *t* test with *p* < 0.05.

## 3. Results

### 3.1. The Impact of Haserpin-e on the Cocoon Production of C. chlorideae

**Figure 1:**
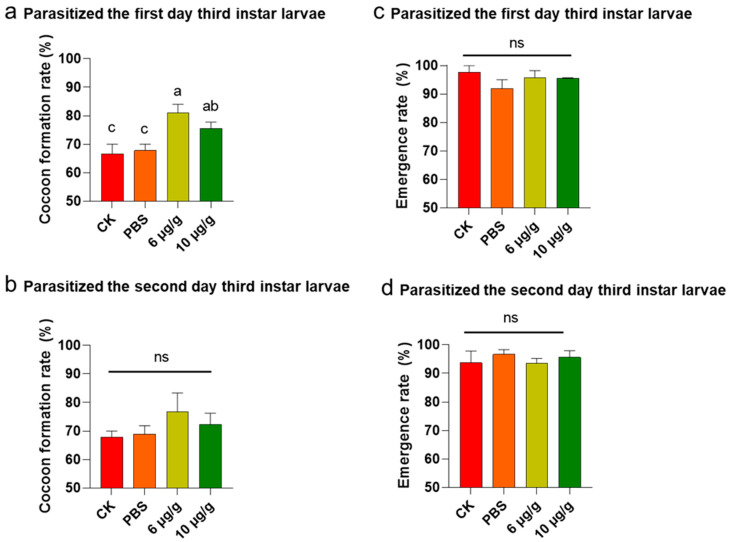
The impact of Haserpin-e supplementation to different developmental stages hosts on the survival capability of F0 *C. chlorideae*. (**a**,**b**) The changes in cocoon formation rate when parasitized third-instar (day 1) larvae and third-instar (day 2) larvae feed on Haserpin-e. (**c**,**d**) The effects on the emergence rate of F0 generation when parasitized the third-instar (day 1) larvae and third-instar (day 2) larvae that feed on Haserpin-e. Values shown are means and standard errors. The data were transformed by arcsine before analysis. Statistically significant differences for experimental comparisons are indicated by different lowercases (*p* < 0.05 level) (based on the Tukey test, SPSS 22.0). The ns indicated no significantly difference among different treatments.

The Haserpin-e protein was purified and dissolved in PBS (Figure S2). The cocoon production significantly increased when *H. armigera* ate Haserpin-e, compared to the blank control and PBS treatment groups (Table 1). Specifically, the cocoon yield of *C. chlorideae* derived from parasitized second-late-stage *H. armigera* showed a significant increase of 7.22% and 4.44% compared to the CK (blank control) and PBS treatment groups when treated with 6 μg/g Haserpin-e, respectively (df = 15, F = 9.31, *p* = 0.002; Table 1). Moreover, the cocoon production exhibited a significant increase of 13.34% and 10.56% compared to the CK and PBS treatment groups under the treatment of 10 μg/g Haserpin-e, respectively (df = 15, F = 9.31, *p* = 0.002; Table 1). The cocoon yield of *C. chlorideae* from hosts parasitized at the third-instar (day 1) Haserpin-e treated *H. armigera* (df = 10, F = 6.609, *p* = 0.022, Figure 1a) was also significantly increased compared to the CK (blank control) and PBS treatment groups, although parasitization of late instar host larvae will result in a decrease in cocooning rate. After *H. armigera* was fed with 6 μg/g Haserpin-e, the cocoon yield increased by 14.45% and 13.34% compared with the CK and PBS treatment groups, respectively (Figure 1a). Similarly, providing *H. armigera* with 10 μg/g Haserpin-e led to cocoon yields that were 8.89% and 7.78% higher compared to the CK and PBS groups, respectively (Figure 1a). The cocoon yield of *C. chlorideae* from hosts parasitized at the third-instar (day 2) *H. armigera*, despite the fact that the effect of Haserpin-e has not yet reached a statistically significant level of impact, the is still an observed increase in cocoon production (Figure 1b, df = 10, F = 0.99, *p* = 0.437). *C. chlorideae* emerged seven days after cocoon formation. For the three stages of parasitism, Haserpin-e did not have any effect on the emergence rate of *C. chlorideae* (Table 1, Figure 1c,d). The second-instar of *H. armigera* is the optimal parasitic stage. We observed the F1 generation of the *C. chlorideae* that parasitized the *H. armigera* at this stage. The *C. chlorideae* of the F0 generation that indirectly contacted Haserpin-e exhibited a higher cocoon production rate; however, this effect was not sustained in the F1 generation (Table 1). A two-way ANOVA revealed a significant interaction effect between the F0 and F1 generations (df = 1, F = 66.39, *p* < 0.0001; Table 1), with the cocoon formation rate of *C. chlorideae* in the F0 generation significantly higher than that in the F1 generation.

### 3.2. The Impact of Haserpin-e on the Life History Traits of C. chlorideae

Haserpin-e significantly affected the length (df = 267, F = 31.42, *p* < 0.0001) and slightly influenced the width of the F0 *C. chlorideae* cocoon, while exerting minimal effects on both dimensions in the F1 generation (Table 1). However, the length and the width of the F0 *C. chlorideae* cocoon is significantly longer than that of F1 *C. chlorideae* cocoon (df = 1, F = 108.25, *p* < 0.0001; df = 1, F = 29.74, *p* < 0.0001; Table 1, Figure S3a). For F0 *C. chlorideae* adults, Haserpin-e significantly affects the tibial length of hind legs (df = 239, F = 7.11, *p* < 0.0001; Table 1, Figure S3b), but not for F1 *C. chlorideae* adults. A two-way ANOVA revealed a significant interaction effect between the F0 and F1 generations (df = 1, F = 52.016, *p* = 0.008; Table 1), and the tibial length of hind legs of *C. chlorideae* in the F0 generation is significantly higher than that in the F1 generation. Ten μg/g Haserpin-e could cause a decrease in the sex ratio in the F0 generation, but it caused a higher mating rate. This effect did not carry over into the F1 generation. A two-way ANOVA revealed a significant interaction effect of mating rate (df = 1, F = 10.312, *p* = 0.004; Table 1) and sex ratio (df = 1, F = 142.10, *p* < 0.0001; Table 1) between the F0 and F1 generations. When treated with Haserpin-e, no significant changes were observed in the male or female survival time for the F0 and F1 generations, and between the F0 and F1 generations. After the adults were dead the mature eggs were recorded, and Haserpin-e did not affect the mature eggs not only in the F0 generation, but also in the F1 generation. And also there were significant interaction effects of mature eggs (dead adults) between the F0 and F1 generations (df = 1, F = 14.495, *p* < 0.0001; Table 1). For understanding the effects on the reproduction of F0 *C. chlorideae*, mature eggs and ovarioles were observed during the reproductive peak of *C. chlorideae* (Table 1, Figures S4 and S5). No adverse effects were detected (Table 1, Figures S4 and S5).

### 3.3. The Effects of Haserpin-e Protein on the Encapsulation of C. chlorideae in H. armigera

**Figure 2:**
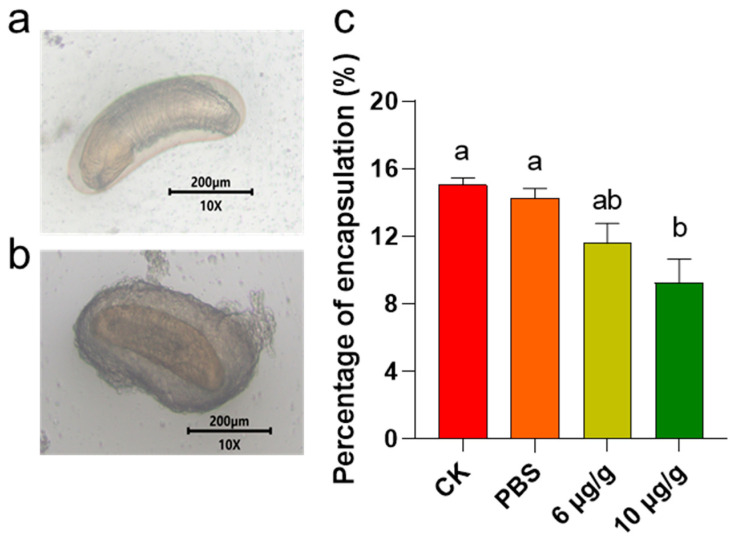
The impact of Haserpin-e on the encapsulation of *H. armigera*. (**a**) Photographs of unencapsulated *C. chlorideae*. (**b**) Photographs of encapsulated *C. chlorideae*. (**c**) Percentage of encapsulation of *C. chlorideae* in *H. armigera*. Values shown are means and standard errors. The data were transformed by arcsine before analysis. Statistically significant differences for experimental comparisons are indicated by different lowercases (*p* < 0.05 level) (based on Tukey test, SPSS 22.0).

Encapsulation is a common response to exogenous invasion. The results indicated that a treatment with 6 μg/g Haserpin-e led to a reduction in the encapsulation of *C. chlorideae* in *H. armigera* by 3.46% and 2.69%, compared to the CK and PBS treatment groups, respectively (Figure 2). Furthermore, administering 10 μg/g Haserpin-e resulted in a reduction in encapsulation by 5.78% relative to the CK group and by 5.01% compared to the PBS group, respectively (df = 11, F = 7.124, *p* = 0.015; Figure 2).

### 3.4. The Effects Haserpin-e Protein on Melanization of H. armigera Larvae Hemolymph In Vitro

**Figure 3:**
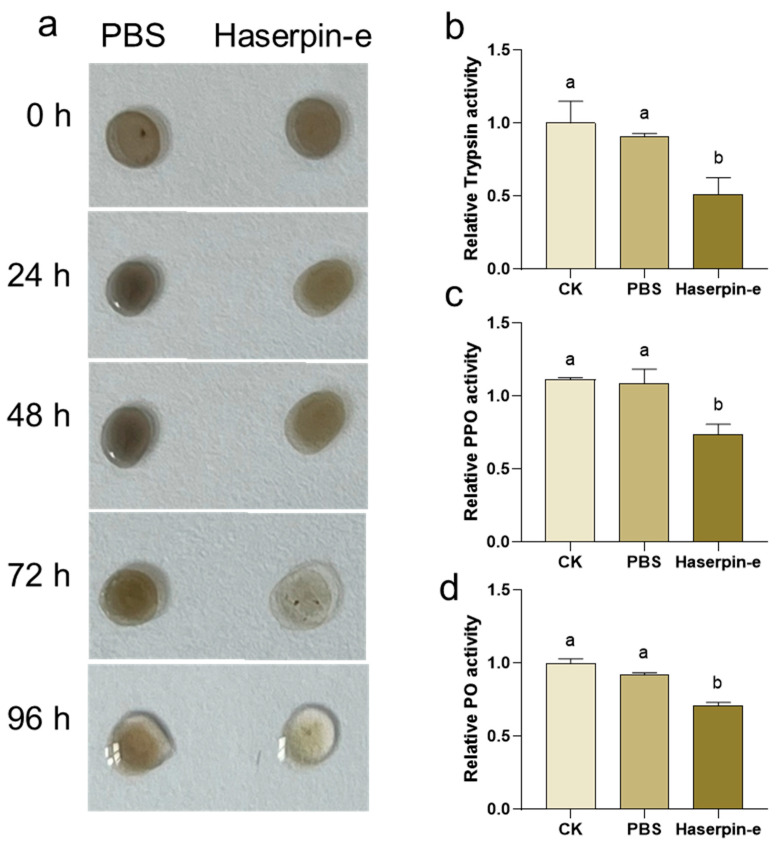
The impact of Haserpin-e on melanization of hemolymph form *H. armigera* larvae. (**a**) Haserpin-e exhibits reactivity with the color of the hemolymph. (**b**–**d**) Haserpin-e modulates the activities of trypsin, polyphenol oxidase (PPO), and phenol oxidase (PO) in the hemolymph. Values shown are means and standard errors. Statistically significant differences for experimental comparisons are indicated by different lowercases (*p* < 0.05) (based on a Tukey test, SPSS 22.0).

Since Haserpin-e acts as a trypsin inhibitor, it can suppress the enzymatic activities of trypsin 38. Moreover, serpin has been reported to play a role in insect melanization upon external invasion [49]. Therefore, the impact of Haserpin-e on the melanization process in *H. armigera* larvae hemolymph in vitro was investigated. When mixed with *H. armigera* larvae hemolymph, Haserpin-e significantly inhibited melanization after 72 h (Figure 3a). Furthermore, the analysis of key enzymes involved in melanization including trypsin, PPO, and PO revealed that Haserpin-e significantly suppressed the enzyme activities of trypsin (df = 10, F = 5.047, *p* = 0.038; Figure 3b), PPO (df = 8, F = 7.529, *p* = 0.019; Figure 3c), and PO (df = 10, F = 40.18, *p* < 0.001; Figure 3d).

### 3.5. The Effects Haserpin-e Protein on Melanization of H. armigera In Vivo

**Figure 4:**
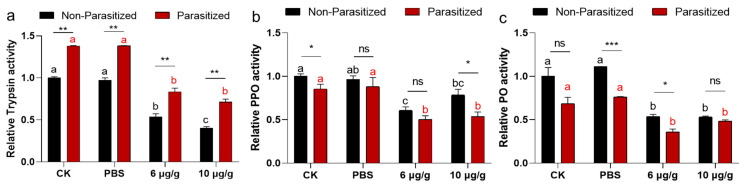
The impacts of Haserpin-e on trypsin (**a**), polyphenol oxidase (PPO) (**b**), and phenol oxidase (PO) (**c**) activities in non-parasitized and parasitized *H. armigera* larvae. Values shown are means and standard errors. Statistically significant differences among different Haserpin-e treatments in no-parasitized or parasitized *H. armigera* larvae are denoted by lowercase letters for *p* < 0.05, based on a Tukey test using SPSS 22.0 software. The statistically significant differences between non-parasitized and parasitized *H. armigera* larvae in each treatment group are represented by different lowercase letters (* *p* < 0.05, ** *p* < 0.01, *** *p* < 0.001, based on *t* test, SPSS 22.0). The ns indicated no significant difference between non-parasitized and parasitized *H. armigera* larvae.

A two-way ANOVA revealed no significant interaction effect between the parasitized status and Haserpin-e treatments in trypsin activities (df = 3, F = 1.333, *p* = 0.6795; Figure 4a). Similarly, parasitized status has independent effects on trypsin activities (df = 1, F = 224.422, *p* < 0.0001; Figure 4a). Haserpin-e treatments have independent effects on trypsin activities (df = 3, F = 202.695, *p* < 0.0001; Figure 4a). The results revealed a significantly higher trypsin activity in parasitized *H. armigera* compared to non-parasitized *H. armigera* in every treatment (Figure 4a). However, Haserpin-e treatment led to suppressed trypsin activities in both parasitized and non-parasitized *H. armigera* (Figure 4a). For the enzyme activities of PPO, there was no significant interaction effect between the parasitized status and Haserpin-e treatments in PPO activities (df = 3, F = 0.975, *p* = 0.8599; Figure 4b). Similarly, parasitized status has independent effects on PPO activities (df = 1, F = 12.168, *p* = 0.0016; Figure 4b). Haserpin-e treatments have independent effects on PPO activities (df = 3, F = 22.779, *p* = 0.0001; Figure 4b). Additionally, Haserpin-e was found to significantly inhibit both parasitized (*p* < 0.0004) and non-parasitized (*p* < 0.0001) *H. armigera*, respectively, with no significant difference between the parasitized and non-parasitized in each treatment (Figure 4b). For the enzyme activities of PO, there was no significant interaction effect between the parasitized status and Haserpin-e treatments in PO activities (df = 3, F = 3.191, *p* = 0.057; Figure 4c). Similarly, parasitized status has independent effects on PO activities (df = 1, F = 35.236, *p* < 0.0001; Figure 4c). Haserpin-e treatments have independent effects on PO activities too (df = 3, F = 41.742, *p* < 0.0001; Figure 4c). In terms of PO, there was a notable decrease in enzyme activity among parasitized *H. armigera* within each treatment group (Figure 4c), and Haserpin-e effectively inhibited PO activities in both parasitized and non-parasitized individuals (Figure 4c).

### 3.6. Haserpin-e Suppresses the Expression of AMPs Expression in H. armigera

**Figure 5:**
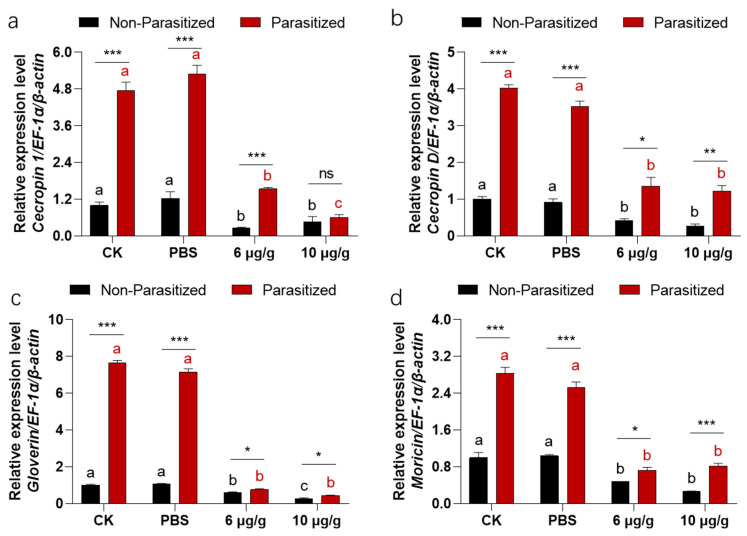
The impacts of Haserpin-e on the expression of *cecropin 1* (**a**), *cecropin D* (**b**), *gloverin* (**c**), and *moricin* (**d**) in non-parasitized and parasitized *H. armigera* larvae. Values shown represent means with standard errors. Statistically significant differences among different Haserpin-e treatments in non-parasitized or parasitized *H. armigera* larvae are denoted by lowercase letters for *p* < 0.05, based on a Tukey test using SPSS 22.0 software. The statistically significant differences between non-parasitized and parasitized *H. armigera* larvae in each treatment group are represented by different lowercase letters (* *p* < 0.05, ** *p* < 0.01, *** *p* < 0.001, based on *t* test, SPSS 22.0). The ns indicated a significantly difference between non-parasitized and parasitized *H. armigera* larvae.

A two-way ANOVA revealed significant interaction effects between the parasitized status and Haserpin-e treatments in cecropin 1 (df = 3, F = 118.51, *p* < 0.0001; Figure 5a), cecropin D (df = 3, F = 38.15, *p* = 0.0.776; Figure 5b), gloverin (df = 3, F = 912.942, *p* < 0.004; Figure 5c), and moricin (df = 3, F = 55.033, *p* = 0.0017; Figure 5d). Similarly, parasitized status has independent effects on cecropin 1 (df = 1, F = 57.77, *p* = 0.0002; Figure 5a), cecropin D (df = 1, F = 451.42, *p* < 0.001; Figure 5b), gloverin (df = 1, F = 2996.12, *p* = 0.0210; Figure 5c), and moricin (df = 1, F = 416.17, *p* < 0.0210; Figure 5d). Haserpin-e treatments have independent effects on cecropin 1 (df = 3, F = 334.32, *p* < 0.0001; Figure 5a), cecropin D (df = 3, F = 103.85, *p* < 0.0002; Figure 5b), gloverin (df = 3, F = 1288.70, *p* < 0.0001; Figure 5c), and moricin (df = 3, F =214.14, *p* < 0.0001; Figure 5d). After being parasitized by *C. chlorideae*, the expressions of cecropin 1 (Figure 5a), cecropin D (Figure 5b), gloverin (Figure 5c), and moricin (Figure 5d) were all significantly increased, repectively. However, upon Haserpin-e administration, the expression of cecropin 1 (Figure 5a), cecropin D (Figure 5b), gloverin (Figure 5c), and moricin (Figure 5d) all were significantly suspressed by Haserpin-e.

## 4. Discussion

**Figure 6:**
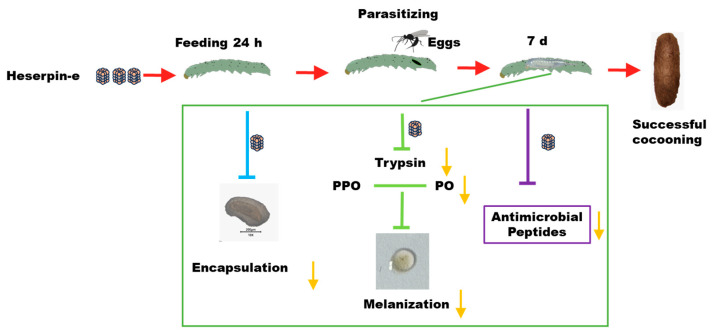
The Haserpin-e derived from *H. armigera* negatively regulates encapsulation, melanization, and AMPs expression in larvae, thereby enhancing large-scale indoor reproduction of *C. chlorideae*.

*C. chlorideae* serves as one of vital biological control agents against Lepidopteran pests due to their significant impact on host populations [1] and high rates of parasitism observed in field studies [5]. To improve the rearing efficiency of *C. chlorideae*, Zhang et al. [49] documented that superparasitism could significantly increase the *C. chlorideae* cocoon production. Here, our study firstly provides a different methodology for large-scale propagation of *C. chlorideae* by feeding the host with Haserpin-e protein to inhibit host’s innate immune responses, which does so by inhibiting melanization, decreasing encapsulation, and reducing the expression of AMPs (cecropin 1, cecropin D, gloverin, and moricin), ultimately enhancing *C. chlorideae* survival (Figure 6). Compared to superparasitism, this method conserves the number of eggs utilized, as regardless of the number of eggs deposited only a single parasitic wasp will fully develop within the host insect. This finding holds significant implications for large-scale indoor reproduction of *C. chlorideae*.

In addition, assessments of life history traits in both F0 and F1 generations revealed that Haserpin-e nearly showed no adverse effect on the growth, development, and reproduction in F0 and F1 generation, except for the size of *C. chlorideae* (Table 1). A significant reduction in *H. armigera* weight was observed when they were fed Haserpin-e (Figure S6). Consequently, it is plausible that the reduced size of F0 and F1 *C. chlorideae* could be attributed to their consumption of thinner *H. armigera*. However, rate of cocoon production, emergence rate, sex ratio, mating rate, the size of *C. chlorideae*, and number of mature eggs (in dead adults) all showed significant interaction effects between the F0 and F1 generations (Table 1). Based on our extensive experience in indoor rearing, it is hypothesized that this phenomenon may be attributed to the degradation of the indoor experimental population of *C. chlorideae*, because there were no changes between the Haserpin-e treatments and the CK (or PBS treatment) group in F0 or F1 generations. Degradation in insect populations under long-term laboratory conditions has been frequently documented in previous studies [50].

By exploring functions and mechanisms of Haserpin-e in *H. armigera* and the cocoon formation promotion process, we observed that Haserpin-e exhibited the ability to reduce encapsulation in *H. armigera* (Figure 2). This result is consistent with the report that the introduction of serpin27A into *D. melanogaster* Meigen larvae with high immune competence significantly impaired their ability to form melanotic capsules around eggs of *L. boulardi* [18]. In addition, this study also found no significant interaction effect between the parasitized status and Haserpin-e treatments in trypsin, PPO, and PO activities, but parasitized status or Haserpin-e treatments have independent effects, respectively, which indicated that both parasitized status and Haserpin-e treatments could inhibit melanization of the host (Figure 3 and Figure 4). These are consistent with previous reports that *C. chlorideae* could inhibit melanization by polydnaviruses [43] and serpin had the functions of inhibiting melanization in many insects [25,26,28,29,30,31,32,33,34,35,36,37]. Although the exact mechanism by which orally administered Haserpin-e reaches systemic immunity in the host remains unclear, many protein fragments expressed by prokaryotes can indeed enter insects and perform their functions. For example, proteins such as cadherin, ALP, and ATP synthase subunit α fragments, when ingested orally by *H. armigera*, enhance the toxicity of Cry1A toxins to *H. armigera* [51,52,53]. This provides evidence of Haserpin-e’s role in regulating encapsulation and melanization, which deepens our understanding of Haserpin-e’s effects.

In addition, this study revealed significant interaction effects between the parasitized status and Haserpin-e treatments in four AMP genes, and showed that parasitized status or Haserpin-e treatments have independent effects on four AMP genes (Figure 5). This phenomenon of up-regulated AMPs overexpression due to parasitism has also been observed in silkworms. In response to *Exorista japonica* Townsend parasitoid attack, the expressions of 13 AMP genes from families such as lysozyme, gloverin, cecropin, enbocin, attacin, and moricin were all up-regulated [54]. AMPs play a crucial role in the humoral defense responses of insects and are negative regulated by serpins [31,55,56]. We discovered that upon administration of Haserpin-e, the expression levels of cecropin 1, cecropin D, gloverin, and moricin were all significantly suppressed by Haserpin-e treatment, and this effect was particularly pronounced in parasitized *H. armigera* (Figure 5), suggesting that Haserpin-e contributes to the survival of *C. chlorideae* by reducing the expression of AMPs induced by parasitism. It is hypothesized that Haserpin-e may directly or indirectly modulate certain antimicrobial peptide synthesis pathways; however, the precise mechanism remains to be elucidated. Our findings here provide additional evidence for the regulatory function of Haserpin-e on AMPs’ expression.

## 5. Conclusions

To the best of our knowledge, this study represents a pioneering approach in promoting the survival of *C. chlorideae* in large-scale indoor reproduction involving endogenous Haserpin-e protein derived from the host *H. armigera*. Additionally, this study expands on the functionalities of Haserpin-e proteins by showing for that Haserpin-e affects the encapsulation, melanization, and AMP expression in *H. armigera*.

## Figures and Tables

**Table 1:** The impact of Haserpin-e supplementation on *H. armigera* larvae on life history traits of *C. chlorideae*.

	Treatments	F0				F1			
Life History Traits		CK	PBS	6 μg/g	10 μg/g	CK	PBS	6 μg/g	10 μg/g
Rate of cocoon production (%)		78.33 ± 3.33 cA	80.83 ± 1.66 bcA	84.99 ± 1.92 bA	91.66 ± 6.38 aA	75.00 ± 1.67 aB	75.00 ± 5.00 aB	71.11 ± 1.92 aB	71.11 ± 1.92 aB
Emergence rate (%)		93.09 ± 3.94 aA	96.23 ± 2.84 aA	91.48 ± 7.87 aA	93.60 ± 2.65 aA	77.99 ± 12.49 aB	79.15 ± 9.91 aB	82.63 ± 5.00 aB	79.93 ± 8.27 aB
Sex ratio (%)		47.72 ± 0.08 abA	46.54 ± 4.59 abA	51.78 ± 8.26 aA	37.27 ± 4.78 bA	26.71 ± 5.29 aB	26.28 ± 2.28 aB	21.05 ± 5.26 aB	20.34 ± 3.03 aB
Mating rate (%)		55.40 ± 16.51 aB	50.92 ± 19.28 aB	58.09 ± 13.62 aB	72.13 ± 6.11 aB	76.38 ± 10.48 aA	75.00 ± 5.00 aA	76.944 ± 14.82 aA	71.95 ± 5.57 aA
Cocoon length (mm)		7.54 ± 0.61 aA	7.69 ± 0.00 a A	6.97 ± 0.67 bA	6.90 ± 0.61 bA	6.77 ± 0.77 aB	6.70 ± 0.43 aB	6.79 ± 0.53 aB	6.60 ± 0.70 aB
Cocoon width (mm)		2.56 ± 0.24 aA	2.51 ± 0.28 aA	2.50 ± 0.24 aA	2.45 ± 0.22 aA	2.40 ± 0.32 aB	2.40 ± 0.23 aB	2.42 ± 0.20 aB	2.31 ± 0.25 AB
Number of mature eggs *		47.62 ± 17.10 aA	53.88 ± 27.06 aA	47.05 ± 17.314 aA	45.88 ± 20.80 aA	40.88 ± 18.55 aB	38.38 ± 16.77 aB	28.33 ± 15.77 aB	35.33 ± 10.29 aB
Tibial length of hind leg (mm)		1.45 ± 0.05 aA	1.44 ± 0.08 aA	1.41 ± 0.07 abA	1.39 ± 0.09 bA	1.37 ± 0.11 aB	1.33 ± 0.11 aB	1.38 ± 0.7 aB	1.35 ± 0.88 aB
Male survival time (day)		15.42 ± 5.67 aA	15.50 ± 5.56 aA	15.88 ± 8.44 aA	14.52 ± 10.43 aA	15.56 ± 9.53 abA	13.71 ± 5.7 abA	12.76 ± 5.7 bA	16.43 ± 7.28 aA
Female survival time (day)		24.62 ± 7.41 aA	25.18 ± 10.43 aA	27.16 ± 12.99 aA	27.88 ± 11.47 aA	22.84 ± 9.31 aA	27.80 ± 7.03 aA	25.10 ± 3.78 aA	25.26 ± 10.61 aA
Ovarioles ^#^		36.06 ± 3.21 a	36.00 ± 2.53 a	36.06 ± 2.01 a	35.33 ± 3.92 a				
Number of mature eggs ξ		82.93 ± 13.48 a	82.46 ± 11.74 a	82.00 ± 18.93 a	82.93 ± 15.64 a				

* Number of mature eggs at the time of adult death. ^#^ Ovarioles in adult on the sixth day (at the reproductive peak). ξ Number of mature eggs in adults on the sixth day (the reproductive peak). Values shown are means and standard errors. Before analysis of variance, all the data were tested under the assumption of normality and homogeneity of variance. Emergence rate data were transformed by arcsine. One-way analysis of variance in the life history traits in each generation and statistically significant differences for experimental comparisons are indicated by different lowercases (*p* < 0.05 level, based on the Tukey test, SPSS 22.0). The two-way ANOVA method was used to statistically analyze the differences in emergence rate, sex ratio, mating rate, ovarioles, and number of mature eggs in adults on the sixth day. The two-way non-parametric ANOVA method was used to statistically analyze the difference in the rate of cocoon production, cocoon length, cocoon width, number of mature eggs at the time of adult death, tibial length of hind leg, and male and female survival time between F1 and F2 generations with SPSS 22.0 at the *p* < 0.05 level of significance. Then, the Tukey method was used for multiple comparisons and the false discovery rate was used to correct the comparison results. Statistically significant differences for experimental comparisons are indicated by a capital letter.

## Data Availability

All the data are in the manuscript and Supplemental Document.

## Supplementary Materials

The following supporting information can be downloaded at: https://www.mdpi.com/article/10.3390/insects16050474/s1, Figure S1: Experiment design and sample sizes in this study; Figure S2: Purification of HaSerpin-e protein; Figure S3: The impact of Haserpin-e on the size of *Campoletis chlorideae* cocoons (a) and the length of their tibiae (b); Figure S4: The impact of Haserpin-e on the ovarin of *Campoletis chlorideae*; Figure S5 The impact of Haserpin-e on the mature eggs of *Campoletis chlorideae e*; Figure S6 The impact of Haserpin-e on the weight of *H. armigera*. (a) Photographs of *H. armigera* under different treatments. (b) The weight of *H. armigera* under different treatments at 72 h. (c) The weight of *H. armigera* under different treatments at 96 h; Table S1: Primers used in this study.
