# Supplementary material for: Enhancing the Survival of Ichneumonid Parasitoid Campoletis chlorideae (Hymenoptera: Ichneumonidae) by Utilizing Haserpin-e Protein to Effectively Manage Lepidopteran Pests
Source: Insects. 2025 Apr 29;16(5):474. doi: 10.3390/insects16050474 (PMC12112269; doi:10.3390/insects16050474)
Supplement: Supplementary file 1 [file insects-16-00474-s001.zip › insects-3530177-supplementary.pdf]

Supplementary Materials

Enhancing the Survival of Ichneumonid Parasitoid *Campoletis chlorideae* (Hymenoptera: Ichneumonidae) by Utilizing Haserpin-e Protein to Effectively Manage Lepidopteran Pests

Liuming Huo [1], Xue Yao [2], Ningbo Zhang [2], Shengyi Wang [2], Sufen Bai [2], Yanmei Wang [1]*, Jizhen Wei [2]*, Shiheng An [2]

1 College of Forestry, Henan Agricultural University, Zhengzhou, 450046, China;

2 College of Plant Protection, Henan Agricultural University, Zhengzhou, 450046, China; 13253830925@163.com (L.H.); yaoxue983@163.com (X.Y.); zhangningbo0109@163.com (N.Z.); wsywsywsy1478@163.com (S.W.); sfbai68@126.com (S.B.); anshiheng@aliyun.com (S.A.)

*Correspondence: wym3554710@163.com (Y.W.); weijizhen1986@163.com (J.W.)

Table S1. Primers used in this study

Gene	Accession number of GenBank	Primer sequences（5′→3′）	Sizes of amplicon (bp)	Amplification efficiency (%)
Cecropin1	GU182916.1	F:AATGAACTTCTCAAGGATA<br>R:TTTAACCTAAAGCTTTGGC	191	95
CecropinD	EU041763.1	F:TGTTTGCTTGGTTCTGGTT<br>R:TTTTCTTCCGAGCTGTCGT	156	100
Gloverin	KT346373.1	F:TTAGCCCTTACGGTGACAG<br>R:ATCCAAGTTGCCTCCCGCTGAT	179	105
Moricin	GU182911.1	F:TGGAAGTAATTAGAATCATG<br>R:TGGCTTGAAGAATGTGTAGA	201	101
β-Actin	HM629442.1	F:CCTGGTATTGCTGACCGTATGC<br>R:CTGTTGGAAGGTGGAGAGGGAA	144	100
EF-1α	U20129.1	F:GCCTGGTACCATTGTCGTCT<br>R:GTAACCACGACGCAACTCCT	154	98

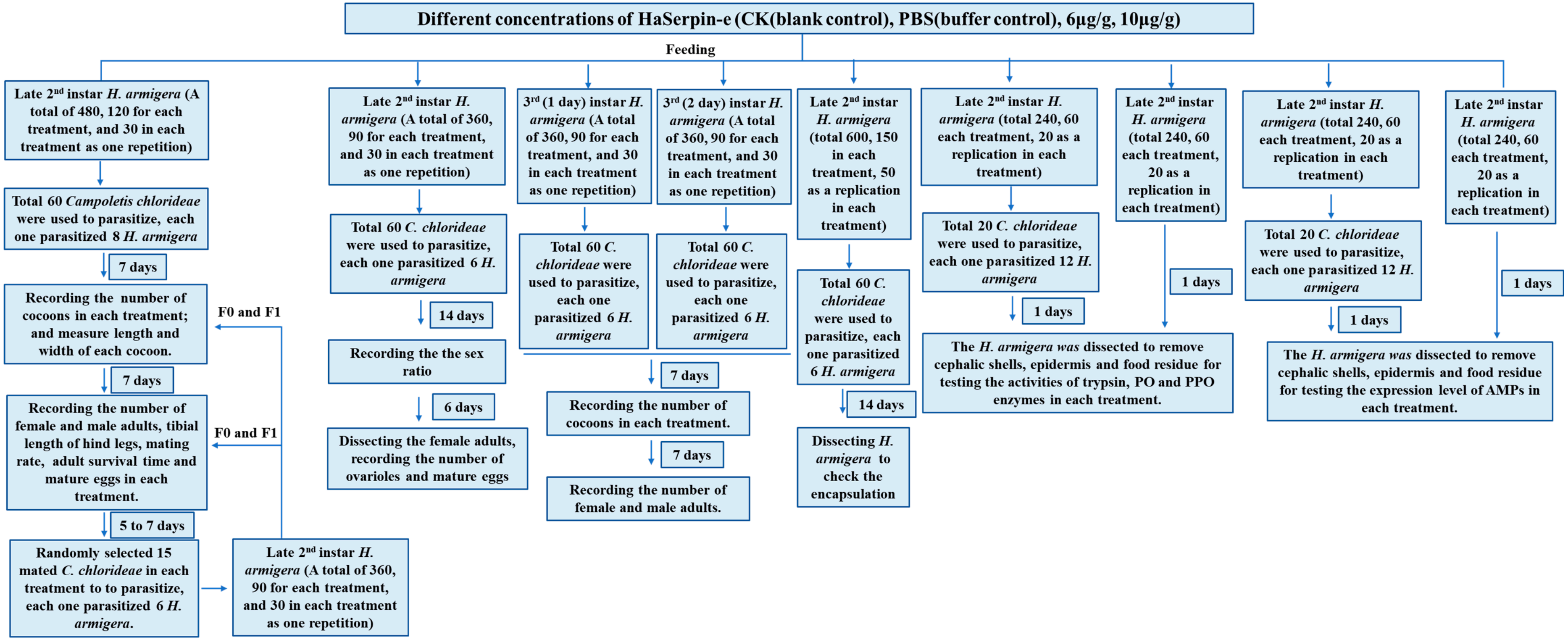

Figure S1. Experiment design and sample sizes in this study.

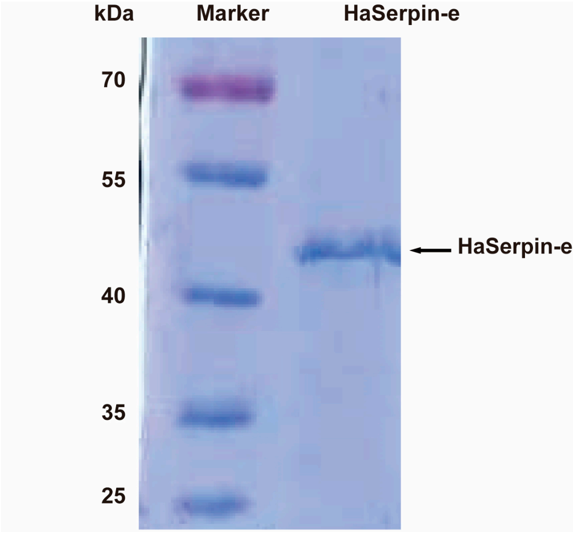

Figure. S2. Purification of HaSerpin-e protein

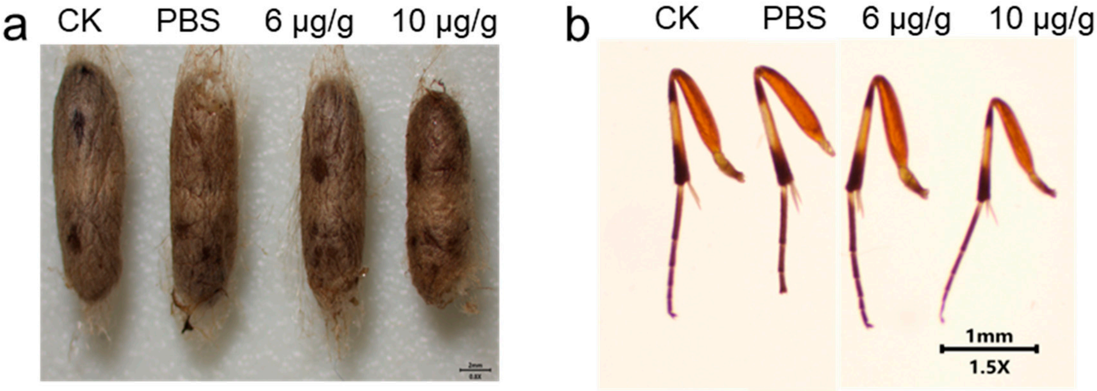

Figure. S3. The impact of Haserpin-e on the size of *Campoletis chlorideae* cocoons (a) and the length of their tibiae (b).

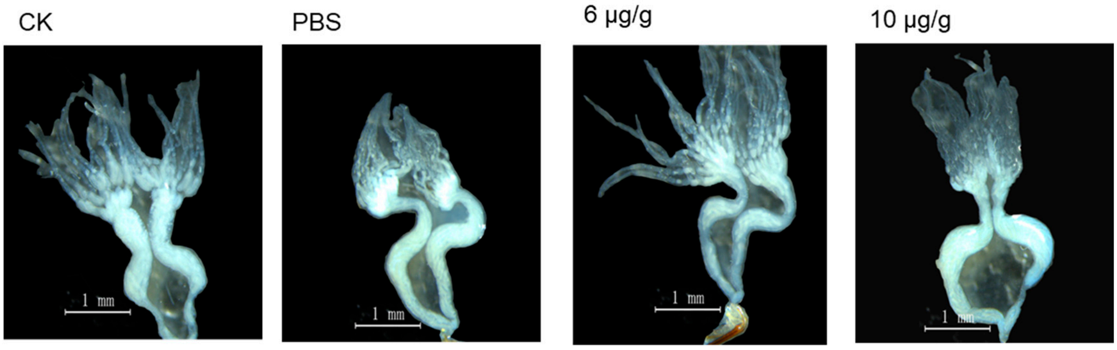

Figure. S4. The impact of Haserpin-e on the ovarin of *Campoletis chlorideae*.

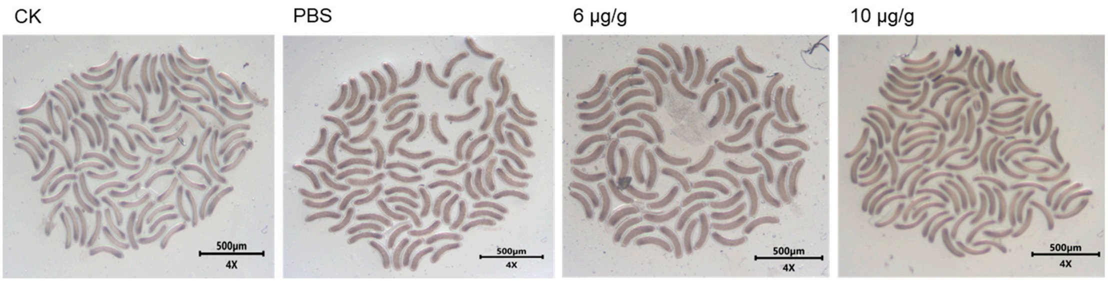

Figure. S5. The impact of Haserpin-e on the mature eggs of *Campoletis chlorideae*.

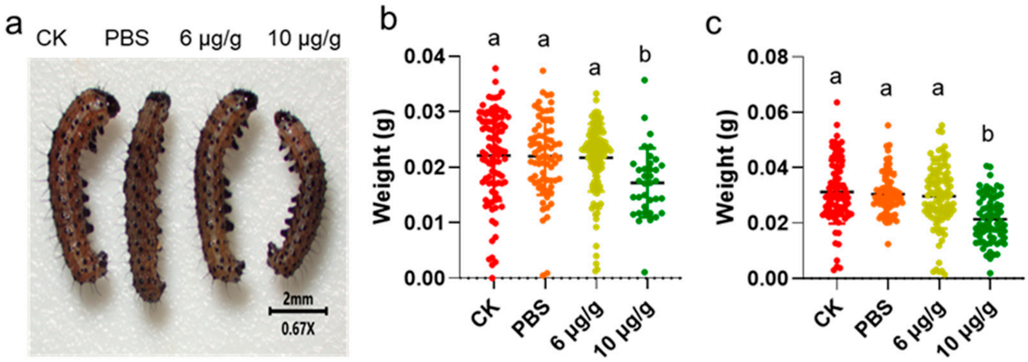

Figure. S6. The impact of Haserpin-e on the weight of *H. armigera*. (a) Photographs of *H. armigera* under different treatments. (b) The weight of *H. armigera* under different treatments at 72 h. (c) The weight of *H. armigera* under different treatments at 96 h. Statistically significant differences for experimental comparisons are indicated by different lowercases (*P* < 0.05 level) (based on Tukey, SPSS 22.0).
